# Supplementary material for: Environmental Factors Drive Chalcid Body Size Increases with Altitudinal Gradients for Two Hyper-Diverse Taxa
Source: Insects. 2023 Jan 10;14(1):67. doi: 10.3390/insects14010067 (PMC9865982; doi:10.3390/insects14010067)

Table S1 Overview of the numbers of analyzed specimens in Pteromalidae and Eulophidae.

Data sources		Pteromalidae		Eulophidae	
		Female	Male	Female	Male
**From the field**	Number	458	316	415	316
	Altitudinal range	277~4337 m		16~4237 m	
	Genera	24		23	
	Total	774		731	
**From the BOLD system**	Number	424	208	579	237
	Altitudinal range	4~2052 m		-21~2028 m	
	Genera	41		49	
	Total	632		816	
**SUM**		1406		1547	

Figure S1 The correlation between seven morphological characteristics of both sexes in Pteromalidae(a) and Eulophidae(b).

(a)Pteromalidae

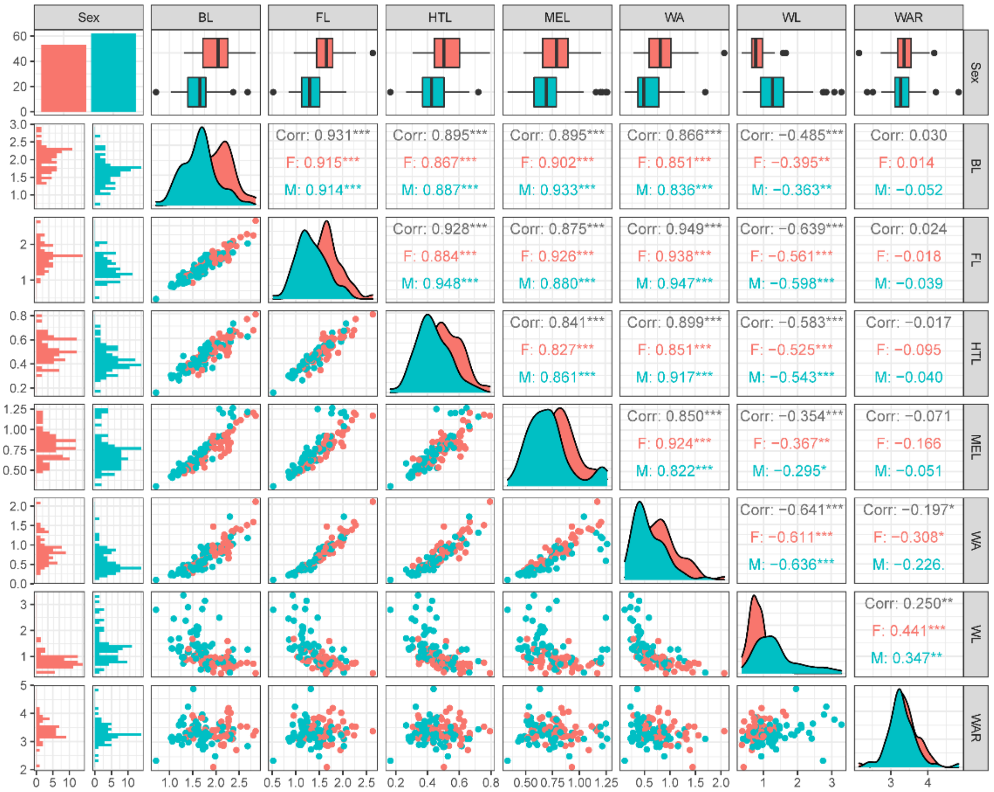

(b)Eulophidae

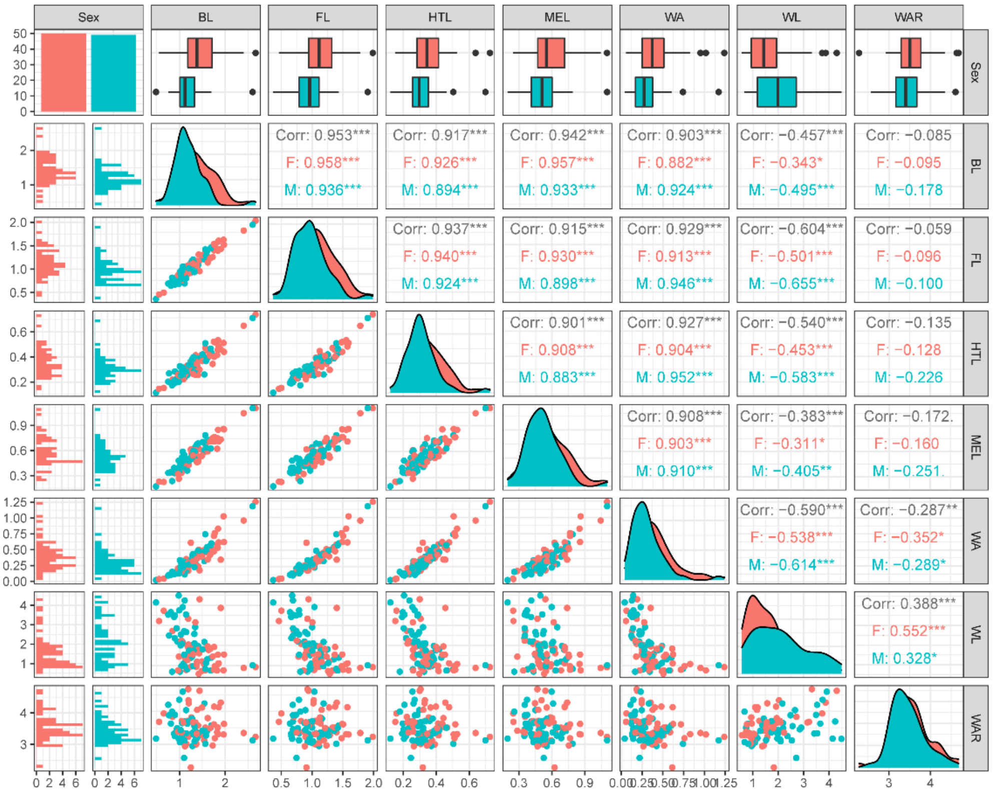

Figure S2 Frequency distribution histogram of the FL of Pteromalidae(a) and Eulophidae(b) from all elevation gradients.

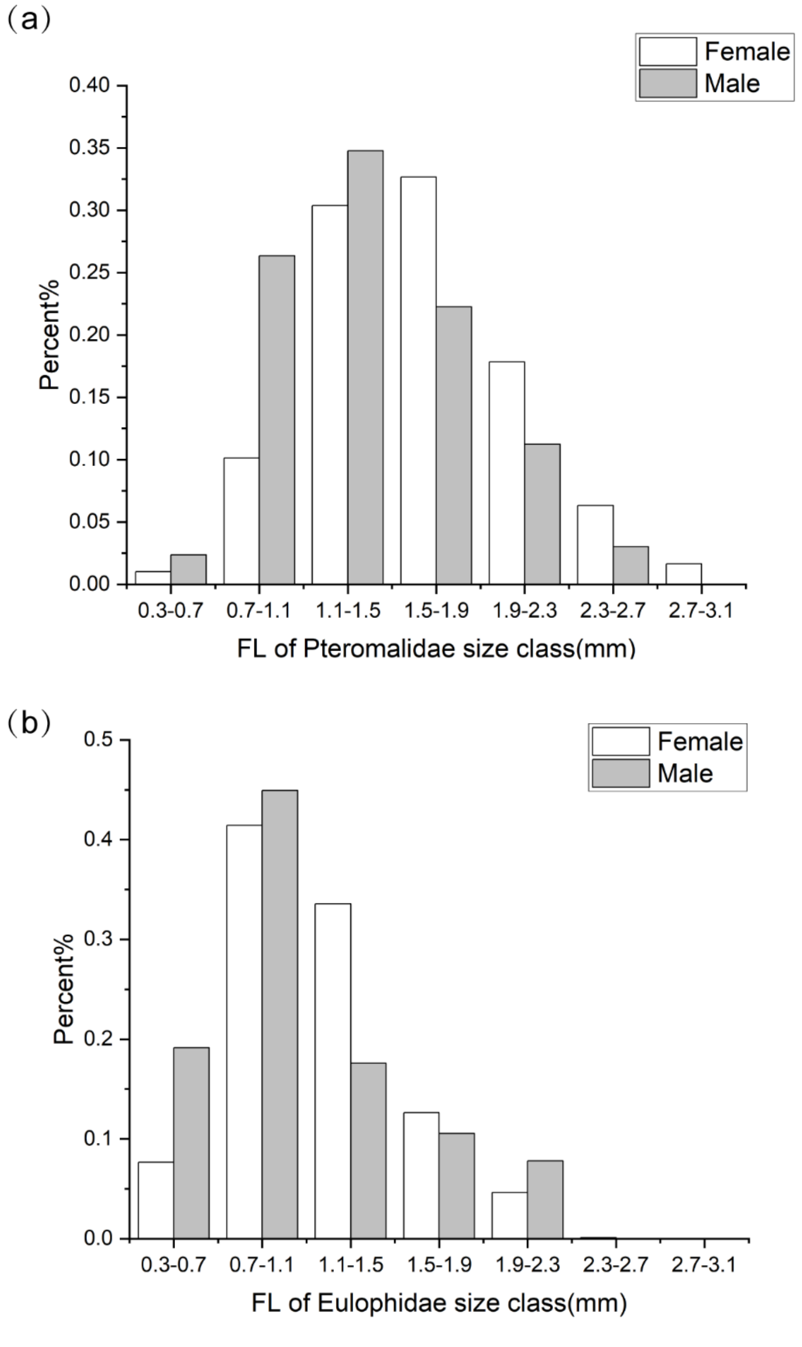

Figure S3 Histogram of the FL of Pteromalidae(a,b) and Eulophidae(c,d) from four elevation ranges.

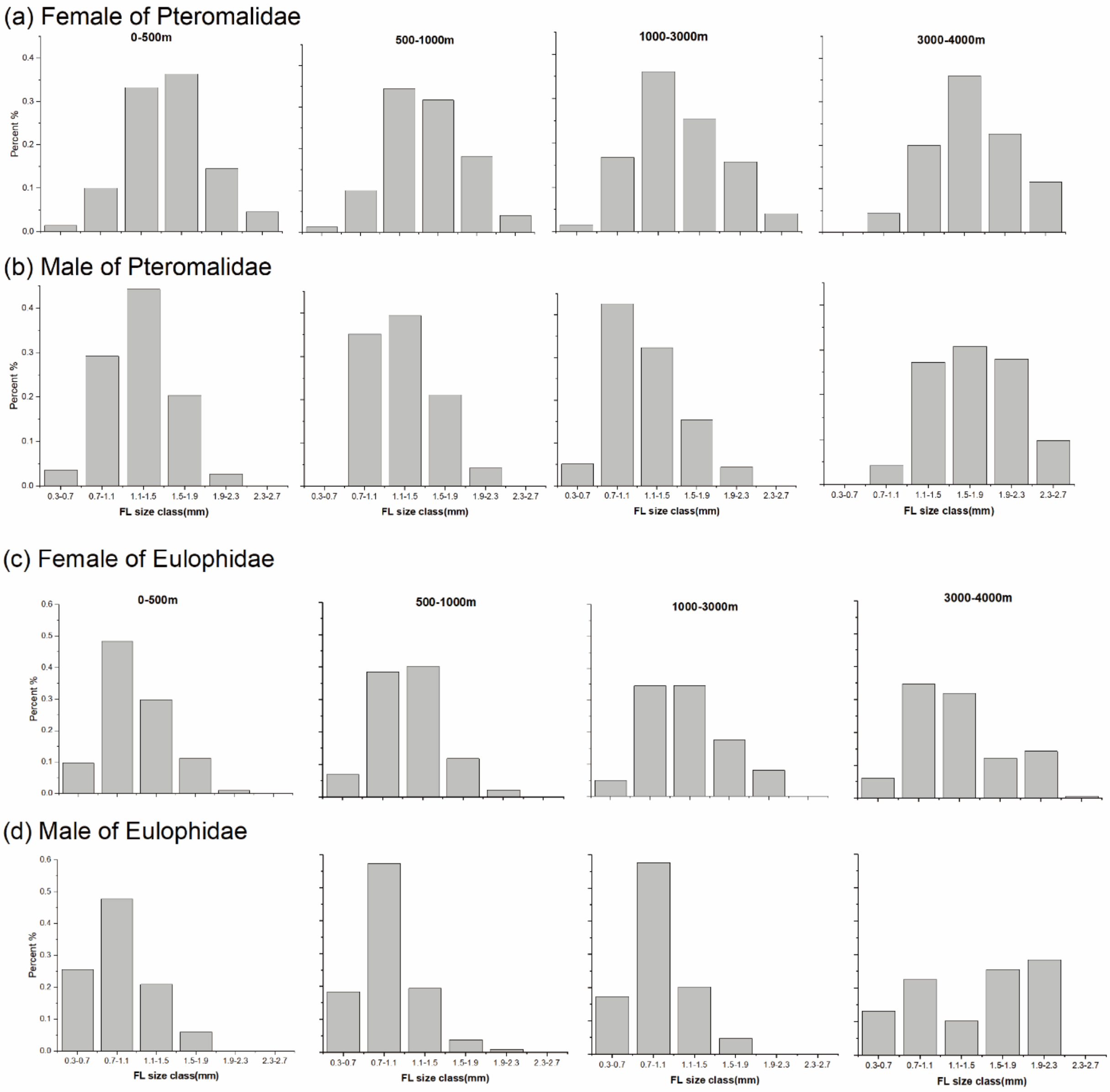

Figure S4 The relationship between forewing length and elevation in six subfamilies from Pteromalidae (a–c) and Eulophidae (d–f).

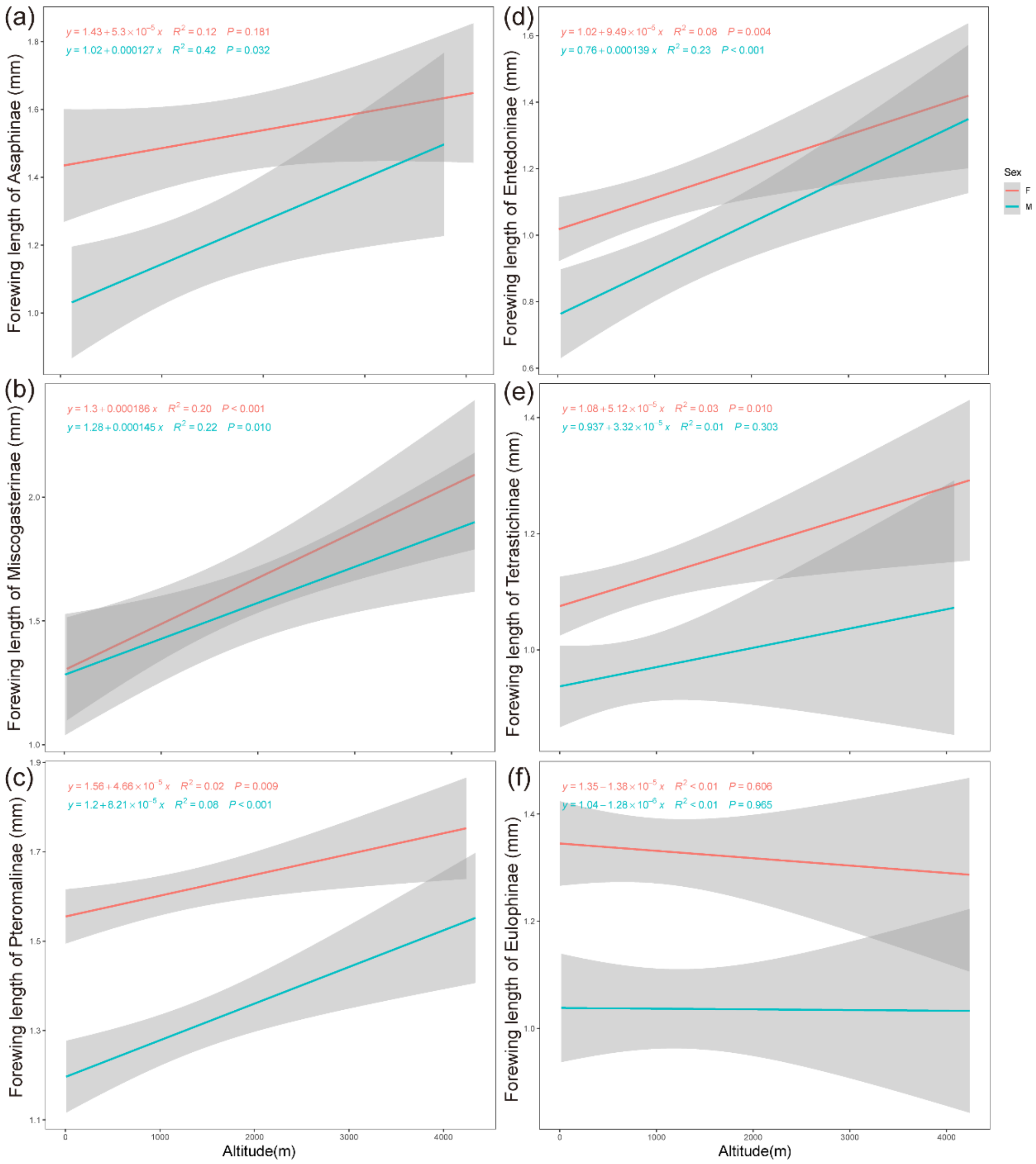

Figure S5 The relationship between forewing length and elevation in eight genera(n ≥ 30 and with wide altitudinal range) of Pteromalidae.

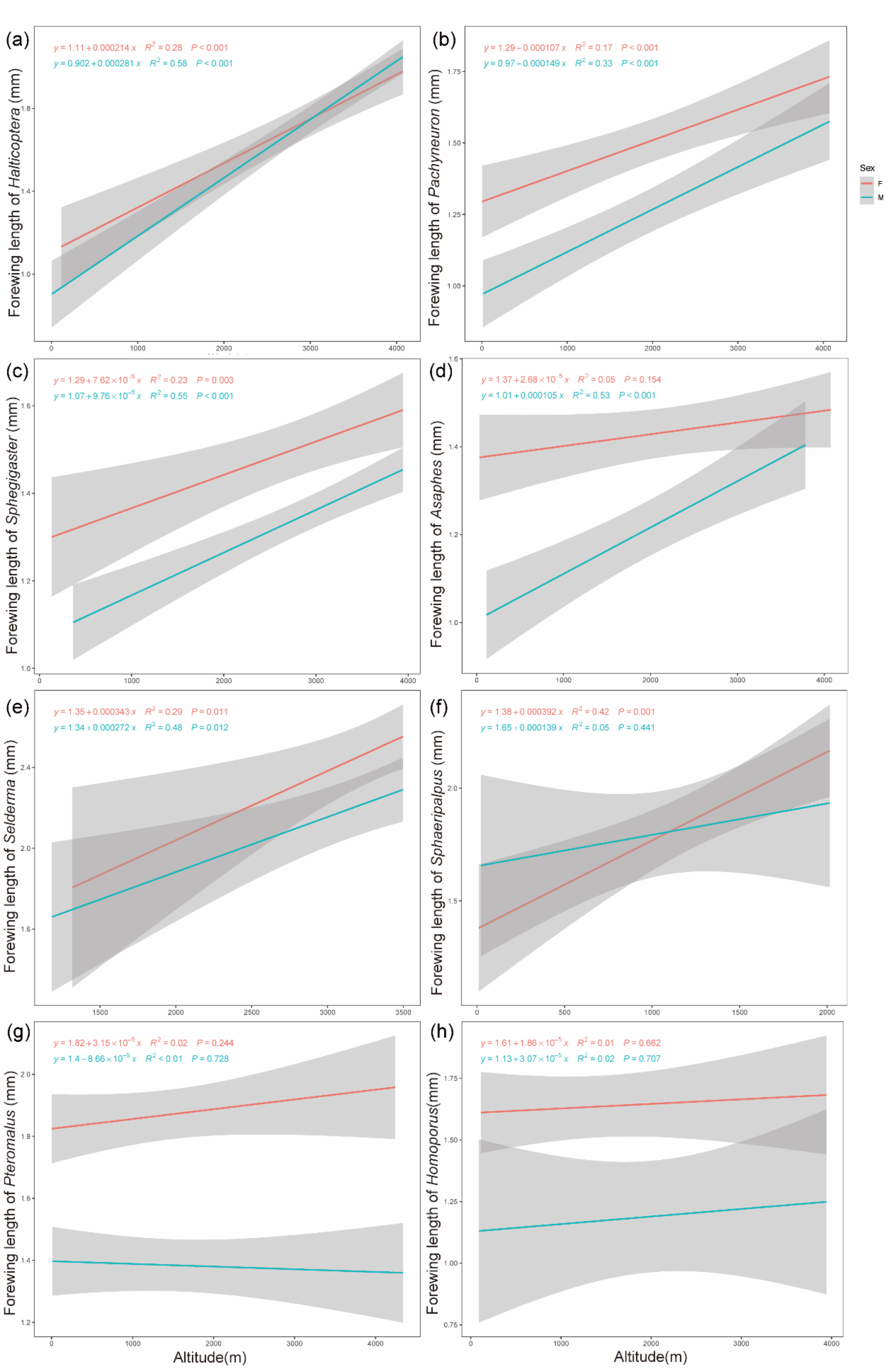

Figure S6 The relationship between forewing length and elevation in eight genera (n ≥ 30 and with wide altitudinal range) of Eulophidae.

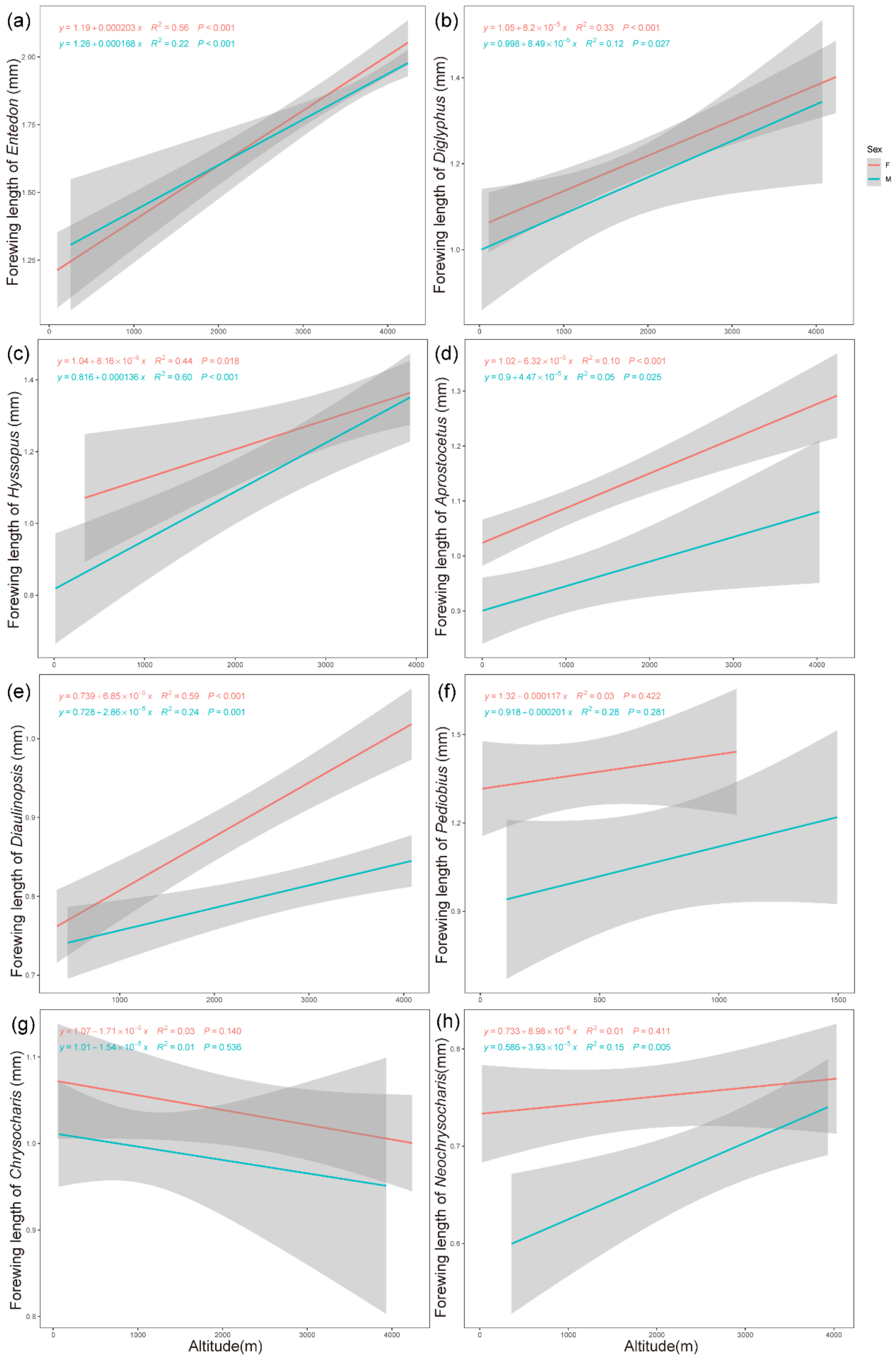

Supplement: Supplementary file 1 [file insects-14-00067-s001.zip › insects-2037604-supplementary/insects-2037604- Supplementary Material/Table and Figures.pdf]
